# Global Transcriptome Profiling of *Salicornia europaea* L. Shoots under NaCl Treatment

**DOI:** 10.1371/journal.pone.0065877

**Published:** 2013-06-25

**Authors:** Jinbiao Ma, Meiru Zhang, Xinlong Xiao, Jinjin You, Junru Wang, Tao Wang, Yinan Yao, Changyan Tian

**Affiliations:** 1 Key Laboratory of Biogeography and Bioresource in Arid Land, Xinjiang Institute of Ecology and Geography, Chinese Academy of Science, Urumqi, China; 2 University of Chinese Academy of Sciences, Beijing, China; 3 College of Resource and Environment Science, Xinjiang University, Urumqi, China; Beijing Institute of Microbiology and Epidemiology, China

## Abstract

**Background:**

Soil salinity is a major abiotic stress that limits agriculture productivity worldwide. *Salicornia europaea* is well adapted to extreme saline environments with more than 1,000 mM NaCl in the soil, so it could serve as an important model species for studying halophilic mechanisms in euhalophytes. To obtain insights into the molecular basis of salt tolerance, we present here the first extensive transcriptome analysis of this species using the Illumina HiSeq™ 2000.

**Principal Findings:**

A total of 41 and 39 million clean reads from the salt-treated (Se200S) and salt-free (SeCKS) tissues of *S. europaea* shoots were obtained, and de novo assembly produced 97,865 and 101,751 unigenes, respectively. Upon further assembly with EST data from both Se200S and SeCKS, 109,712 high-quality non-redundant unigenes were generated with a mean unigene size of 639 bp. Additionally, a total of 3,979 differentially expressed genes (DEGs) were detected between the Se200S and SeCKS libraries, with 348 unigenes solely expressed in Se200S and 460 unigenes solely expressed in SeCKS. Furthermore, we identified a large number of genes that are involved in ion homeostasis and osmotic adjustment, including cation transporters and proteins for the synthesis of low-molecular compounds. All unigenes were functionally annotated within the COG, GO and KEGG pathways, and 10 genes were validated by qRT-PCR.

**Conclusion:**

Our data contains the extensive sequencing and gene-annotation analysis of *S. europaea*. This genetic knowledge will be very useful for future studies on the molecular adaptation to abiotic stress in euhalophytes and will facilitate the genetic manipulation of other economically important crops.

## Introduction

Along with environmental deterioration and anthropogenic influence, soil salinity is one of the major abiotic stresses in plant and crop systems, causing yield losses of primary crops worldwide [1], [2]. Much efforts have been made to enhance salt tolerance of economically important plants for future agricultural demand [3]. Many salt stress-induced genes are part of a complicated cascade of molecular networks and are thought to play important roles in responding to environment stress [4], [5]. These genes are involved in many cellular and physiological processes such as signal perception and transduction [6], [7], photosynthesis, energy metabolism [8], membrane trafficking and protein biosynthesis, folding and decay [9], [10]. The isolation and identification of these functional genes, using the tools of molecular biology, is necessary for understanding the mechanisms of salt tolerance in plants [11]–[13]. Several essential genes involved in different molecular processes are reportedly involved in the plant response to salt stress, including AtHKT1;1 [14], [15], PutHKT2;1 [16], AtSOS1 [17], [18], AtNHX1 [19], Ca^2+^/calmodulin-dependent protein phosphatase [20], flavoprotein AtHAL3 [21], plasma membrane (PM) H^+^-ATPase and transcription factor Alfin1 [22], [23]. However, only a few salt-tolerance genes have been previously identified, and most of the studies on plant salt tolerance have focused on model plants such as Arabidopsis, rice and tobacco. Research on salt-tolerant genes in halophytes is scarce, even though some euhalophytes have the specific ability to cope with high levels of salinity using unique mechanisms [24]–[30].

*Salicornia europaea* L., one of the most salt-accumulating euhalophytes, can withstand concentrations of more than 1,000 mM NaCl in the soil and is widely distributed in coastal and inland salt marshes around the world [24], [31]. It is an important species for soil desalination because it hyper-accumulates salt in weakly saline soil, with salt reaching 50% of the dry weight of shoots. In contrast to glycophytes, which are negatively affected by increasing salinity beyond a threshold of approximately 50 mM, *S. europaea* survives in a high saline habitat. The optimal salinity for maximum growth is in the range of 100 to 200 mM, and its growth is significantly hindered if the soil salinity is outside this range [31]. An ion transport assay performed in the presence of various salts has revealed that Na^+^, Cl^−^, K^+^ and Ca^2+^ were absorbed and distributed into the tissue of *S. europaea*. The root K^+^ content reached 90 meq l^−1^, while in the soil, it never exceeded 5 meq l^−1^. The Na^+^, Cl^−^ and Ca^2+^ concentrations were generally higher in the shoots than in the roots [32]. Previous studies have determined that the fresh and dry weight of the plant reached the highest values at optimal salinity, and superoxide dismutase (SOD), catalase (CAT) and guaiacol peroxidase (GPX) activities also increased at higher salinities [25], [33]. Recently, SePSY (phytoene synthase gene) [34], SeNHX1 (Vacuolar Na^+^/H^+^ antiporters) [35], [36], SeLCY (beta-lycopene cyclase gene) [37] and SeCMO (choline monooxygenase) [38] were isolated from *S. europaea*, and it was shown that the expression of these genes was correlated to the salt concentration. However, information concerning gene expression and regulation as they relate to salt adaptation in *S. europaea* is scarce [39], [40]. Previously, it has been laborious and time-consuming to identify and characterise the genes for salt adaptation or tolerance in *S. europaea* because transcriptomic and genomic data for *S. europaea* were unavailable in public databases. For example, no genome and only fourteen EST sequences were available at the time of publication. In halophytes, large numbers of cDNA fragments from several typical species were isolated from cDNA and SSH libraries [41]–[43], but those sequences could not provide global transcriptome information for the species. Only limited studies of the transcriptome of salt-treated halophytes have been reported thus far. This lack of information is a significant obstacle to our understanding of the molecular mechanisms for salt adaptation in halophyte species and impedes the exploitation of halophytes for the restoration of saline soil.

To obtain novel insights into the molecular basis of salt adaptation in *S. europaea*, two-group *de novo* assembly data were generated from Illumina sequencing of shoot samples for salt-treated or untreated plants. Transcriptome changes resulting from salt treatment were also compared. Our objective was to uncover and characterise a core set of salt stress-related transcripts.

## Materials and Methods

### Plant material and growth conditions

*S. europaea* seeds were collected from saline soil in Fukang, Northwestern China. Seeds were sown in plastic pots (12×12 cm) filled with sands irrigated with tap water. After germination, seedlings were maintained in a greenhouse with a day/night thermoperiod of 25/20°C, a photoperiod of 16 h, relative humidity of 50±10% and weekly irrigation with half-strength hoagland nutrient solution. Two months later, the plants were irrigated with NaCl solutions with concentrations of 0, 10, 200, 400 or 800 mM NaCl. The shoots were harvested at 72 h after salt treatment and frozen immediately in liquid nitrogen for the extraction of total RNA.

### cDNA library preparation and Solexa sequencing for transcriptome analysis

Total RNA was extracted from *S. europaea* shoots treated with 0 mM (SeCKS) and 200 mM (Se200S) NaCl using the QIAGEN RNeasy Plant Mini kit (Qiagen) according to the manufacturer's protocol. The RNA samples were used to construct two tissue-specific cDNA libraries for RNA sequencing and transcriptome analysis. According to the manufacturer Illumina's instructions, poly (A)^+^ RNA was isolated from 20 μg total RNA using oligo (dT) magnetic beads. Fragmentation buffer was added to break the mRNA into short fragments. Using these short fragments for templates, random hexamer-primers were used to synthesise first-strand cDNA. Second-strand cDNA was synthesised using buffer, dNTPs, Rnase H (Invitrogen) and DNA polymerase I (New England Biolabs). The resulting short fragments were purified using the QiaQuick PCR extraction kit and then resuspended in EB buffer for end repair and poly(A) addition. The short fragments were then joined to sequencing adapters. Following agarose gel electrophoresis, suitable fragments were selected for PCR amplification. The libraries could then be sequenced using the Illumina HiSeq™ 2000.

### *De novo* assembly of sequencing reads and sequence clustering

Following cDNA library sequencing, high-quality clean reads were picked out from the raw reads of each library following removal of reads containing adaptor sequences, reads with an N (unknown bases in a read) percentage higher than 5% and low-quality reads (>50%of the bases with a quality score Q-value ≤5) using perl scripts. Transcriptome *de novo* assembly was carried out with the short-reads assembly programme Trinity [44]. We first combined reads with a certain length of overlap to form contigs with a zero N value (no unknown bases). Then, the reads were mapped back to contigs with paired-end reads. This approach detected contigs from the same transcript as well as the distances between these contigs. Next, Trinity connected the contigs using N to represent unknown sequences between each contig pair to form scaffolds. Finally, unigenes were generated with zero N values in the sequence that could not be extended on either end. In the final step, BLASTX alignment (e value <0.00001) between the recovered unigenes and protein databases such as nr, Swiss-Prot, KEGG (Kyoto Encyclopedia of Genes and Genomes) and COG (Clusters of Orthologous Groups) was performed, and the best aligning results were used to determine the sequence direction of the unigenes. If the results from different databases conflicted with one another, a priority order of nr, Swiss-Prot, KEGG and COG was followed when deciding the unigene sequence direction. When a unigene was unaligned in all of the databases, the programme ESTScan was used to decide its sequence direction [45]. For unigenes with verified sequence directions, we provided their sequences in the 5′ to 3′ orientation; for those without direction, we provided their sequences as determined by the assembly software. All the obtained data were submitted to DDBJ/EMBL/GenBank database.

### Unigene functional annotation

The individual unigenes were analysed by searching the protein databases nr, Swiss-Prot, KEGG and COG (e-value<0.00001) with the BLASTX algorithm (http://www.ncbi.nlm.nih.gov/) to retrieve functionally annotated proteins with the highest sequence similarity to the unigenes on our list. The COG database was used to predict and to classify possible functions of our unigenes. The KEGG pathways database was used for inner-cell metabolic pathways annotation and to determine the potential complicated biological behaviours of the genes on our list. The Blast2GO programme was used to understand the GO functional annotations derived from the molecular function, cellular location and biological processes of all of our unigenes [46].

### Differential gene expression of*S. europaea* between salt-free and salt-treated shoots

To determine the DEGs (differentially expressed genes) between SeCKS and Se200S, gene expression level analysis was performed using the RPKM (Reads per kb per million reads) method and the formula RPKM = 10^6^C/10^−3^NL, where C is the number of reads that uniquely align to a unigene, N is the total number of reads that uniquely align to all unigenes, and L is the base number in the Coding sequence (CDS) of a unigene [47]. The RPKM method eliminates the influence of different gene lengths and sequencing levels on the calculation of gene expression. Therefore, calculated gene expression can be directly used for comparing the differences in gene expression between samples. We compared the transcriptome profiles of SeCKS and Se200S for *S. europaea* to detect DEGs between both samples using the statistical method FDR (False discovery rate) and the ratio of RPKMs for the two samples (FDR ≤0.001 and |log_2_^ratio^| ≥1). DEGs were then used to carry out GO functional and KEGG Pathway analysis.

### RT-PCR and qRT-PCR used to validate and analyse the unigenes

RNA was extracted from the shoots and roots of 0, 10, 200, 400, and 800 mM NaCl-treated plants. cDNA was synthesised from 2 μg of RNase-free, DNaseI-treated total RNA with 500 ng of 18 mer oligo-dT primers and M-MLV reverse transcriptase (Promega). The primers for RT-PCR and quantitative RT-PCR were designed using the sequences determined for the differentially expressed unigenes using the programme Primer 5.0. The α-tubulin gene was used as an endogenous control. Sequences of the primers are given in Additional File S1. Quantitative RT-PCR was performed using the CFX96 real-time PCR detection system (Bio-Rad), a 25-µl reaction system and the SYBR Premix Ex Taq Kit (TaKaRa Corp. Beijing, China) according to the manufacturer's protocol. The following cycling programme was used: 95°C for 60 sec, followed by 40 cycles of 10 sec at 95°C, 30 sec at 55°C and 30 sec at 72°C. All products were subjected to melting curve analysis between 55°C and 95°C to determine the specificity of the PCR reaction. The relative quantitative method (^ΔΔ^Ct) was used to evaluate the quantitative variation.

## Results and Discussion

A number of researchers have proven that transcriptome sequencing of RNA/cDNA provides a rapid and cost-effective way to generate whole-transcriptome sequences from many types of biological samples and to identify differentially expressed genes. In particular, transcriptomes from a number of plant species, including *Arabidopsis thaliana*, rice and wheat, were investigated for various purposes [48]–[50]. To the best of our knowledge, although *S. europaea* is one of most salt-tolerant halophytes, only a limited numbers of genes from this organism have been cloned and characterised [24], [51]. Transcriptome sequencing of *S. europaea* is an important step for gene identification and the elucidation of the molecular mechanisms underlying salt adaptation and hyper-accumulation in *S. europaea*.

### *De novo* assembly and quantitative assessment of Illumina ESTs

**Figure 1:**
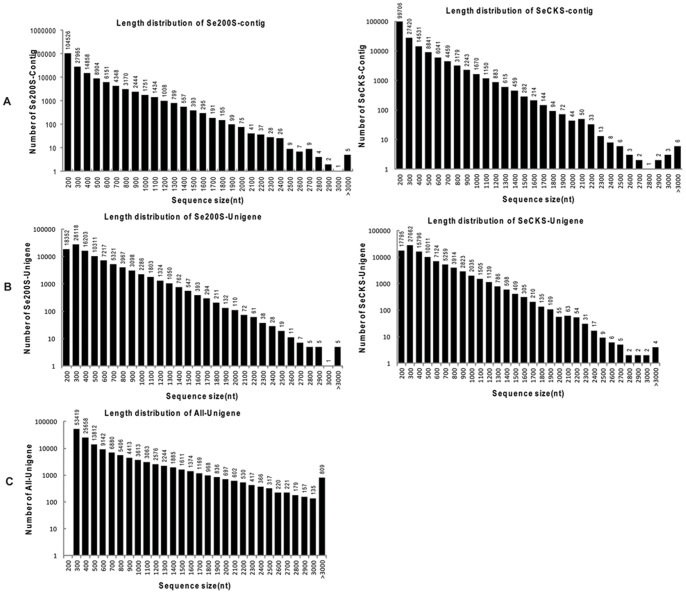
Size distribution of the contigs and unigenes generated by***de novo*** **assembly.** (A) Size distribution of contigs. The x-axis represents contig size, and the y-axis represents numbers of contigs of a certain length. (B) Size distribution of unigenes. The x-axis represents unigene size, and the y-axis represents the number of unigenes with a certain length. (C) Size distribution of All-Unigenes. The x-axis represents All-Unigenes size, and the y-axis represents the number of All-Unigenes with a certain length.

As a first step to gain insight into transcriptional regulation of *S. europaea* adaptation to saline habitats, we used the RNA-Seq technique to generate two tissue-specific whole-transcriptome profiles of *S. europaea* shoots treated with either 0 mM or 200 mM NaCl. After cDNA enrichment and Illumina sequencing, low quality and adapter sequences were eliminated. A total of 41 million and 39 million clean reads were generated from the SeCKS and Se200S shoot cDNA libraries with 97.21 and 97.24% Q20 percentages, respectively. The total length of the reads was 7.21 gigabases (GB). The reads from two libraries were clustered into 172,174 and 179,292 contigs by the programme Trinity. Contig sizes ranged from 100 bp to 3,000 bp, and the average contig sizes were 267 bp for SeCKS and 271 bp for Se200S (Figure 1A). To reduce sequence redundancy, all contigs were further assembled into 97,865 and 101,751 unigenes in the SeCKS and Se200S cDNA libraries, with 424 bp and 438 bp average lengths, respectively, and the size distribution ranged from 200 bp to 3,000 bp (Figure 1B). Unigenes from SeCKS and Se200S were deposited at DDBJ/EMBL/GenBank as the TSA accession GAHZ00000000 and GAIA00000000 respectively. To acquire the longest non-redundant sequences possible, unigenes from SeCKS and Se200S were further assembled, and 109,712 non-redundant unigenes, termed All-Unigene, were generated. The All-Unigene size distribution demonstrated that most sequences (64,411; 58.71%) were less than 500 bp in length; 23.66% (25,961) were between 500 and 1,000 bp; 16.89% (18,532) were between 1,000 and 3,000 bp; and 0.74% (808) were more than 3,000 bp (Figure 1C, table 1). The unigene size distribution revealed that shorter fragments were reduced and longer fragments were generated as a result of further assembly. All of these unigene sequences can be accessed in Additional File S1: Additional File S2.z01, Additional File S2.z02 and Additional File S2.

**Table 1:** Summary of sequencing and assembly results.

sample	number/percent	100–200nt	200–300nt	300–400nt	400–500nt	> = 500nt	N50	Mean	No.	Length (nt)
Se200S-Contig	number	104,526	27,965	14,858	8,904	23,039	372	271	179,292	48,616,772
	percent	58.30%	15.60%	8.29%	4.97%	12.85%				
SeCKS-Contig	number	99,706	27,420	14,531	8,841	21,676	363	267	172,174	45,962,606
	percent	57.91%	15.93%	8.44%	5.13%	12.59%				
Se200S-Unigene	number	18,352	28,118	16,203	10,311	28,767	547	438	101,751	44,551,677
	percent	18.04%	27.63%	15.92%	10.13%	28.27%				
SeCKS-Unigene	number	17,795	27,662	15,796	10,011	26,601	523	424	97,865	41,490,134
	percent	18.18%	28.27%	16.14%	10.23%	27.18%				
All-Unigene	number	0	34,417	18,808	11,186	45, 301	780	636	109,712	69,795,900
	percent	0	31.37%	17.14%	10.20%	41.29%				

N50: 50% of the assembled bases were incorporated into sequence with N50 length or longer.

Mean: Mean length of assembled sequences.

To confirm the sequencing quality of our transcriptome data and sequence assembly, 18 unigenes were randomly selected for RT-PCR analysis. In this analysis, 13 out of 18 primer pairs amplified a product of the expected length (Additional File S3). The unigene sequences were then confirmed by sequencing the PCR products.

### Functional annotation and analysis

**Figure 2:**
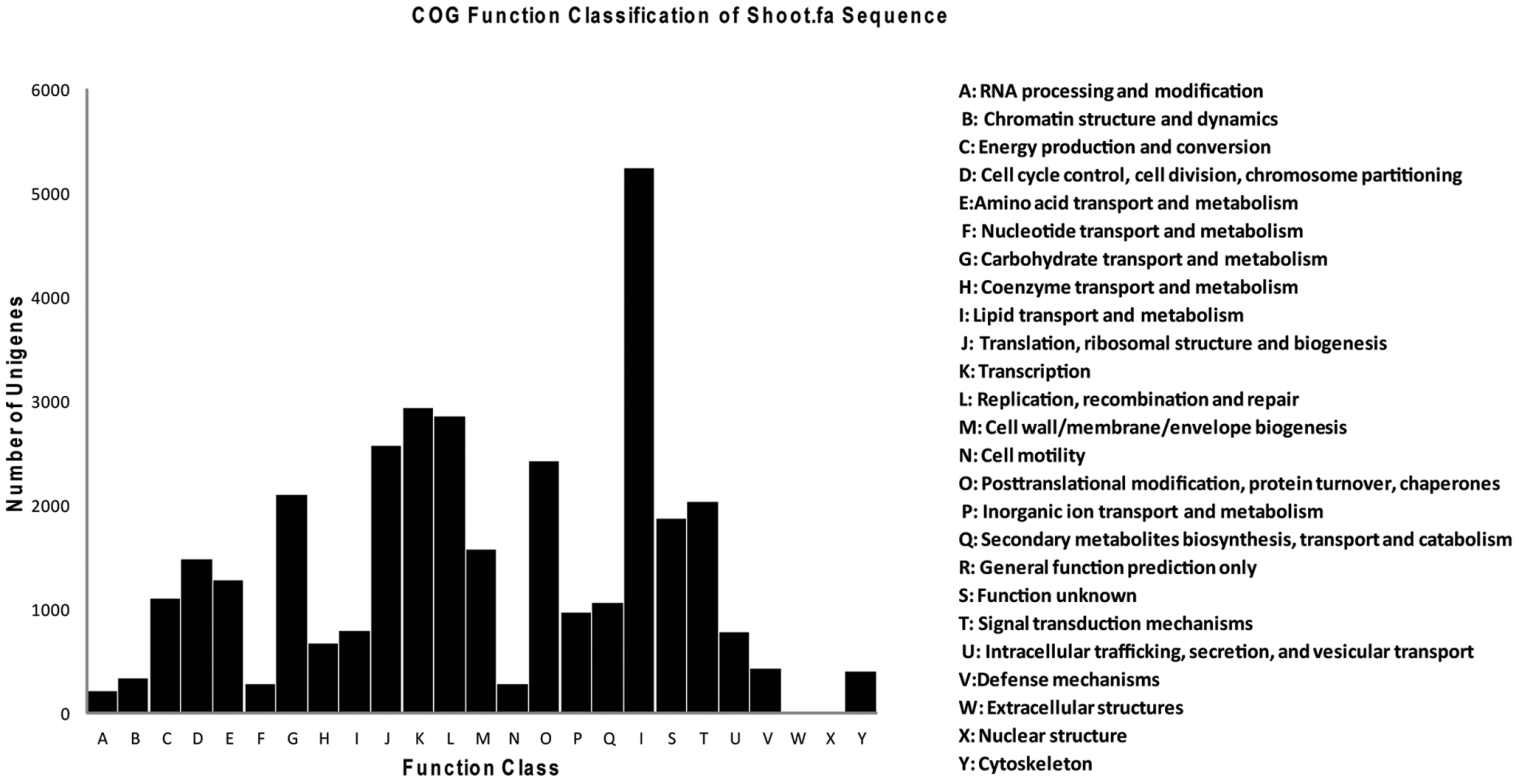
Histogram presentation of COGs classification. A total of 33,636 (16.2%) unigenes out of the All-Unigenes set were aligned to the COG database and classified into 25 functional-categories.

**Figure 3:**
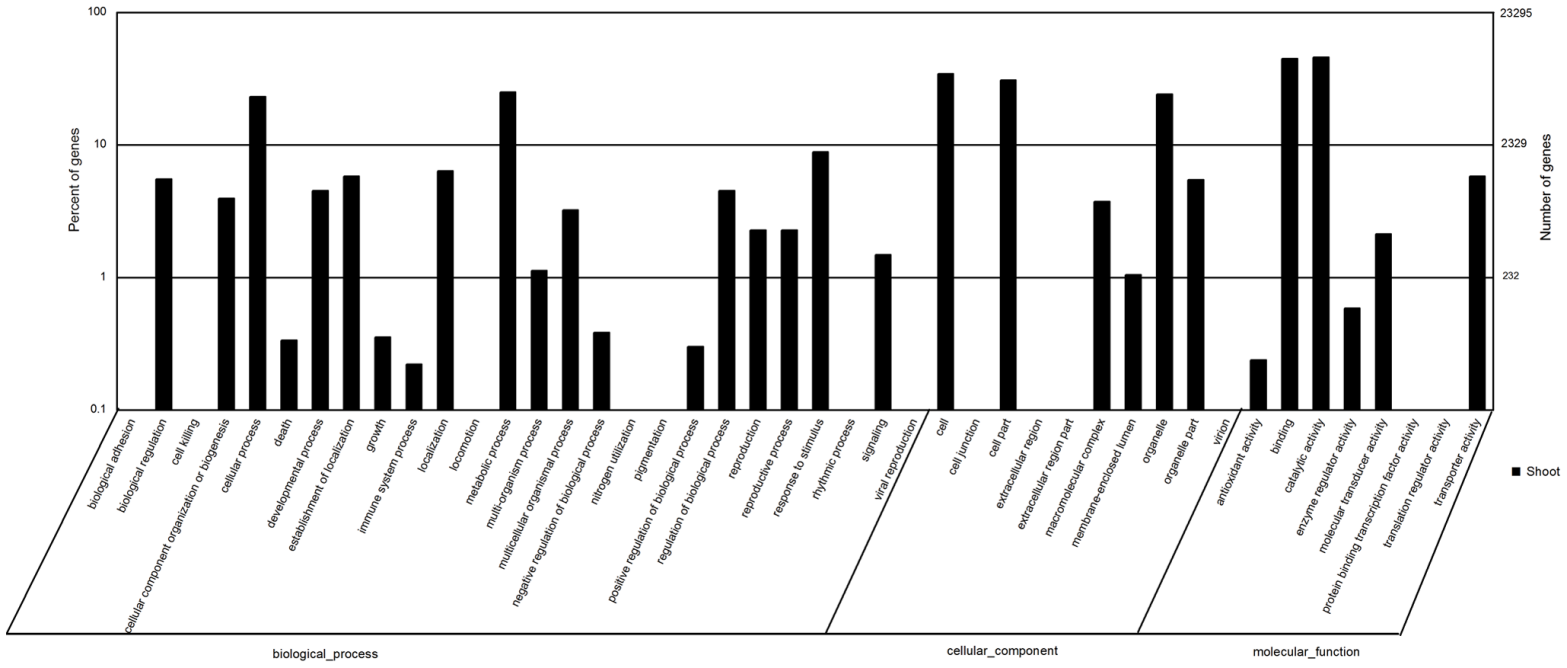
Histogram presentation of GO classification. A total of 35,268 (32.15%), 43,713 (39.84%) and 21,334 (19.45%) unigenes of *S. europaea* were classified into 44 terms from three ontologies involved in biological processes, cellular components and molecular function, respectively. The *right y axis* indicates the number of genes in a category. The *left y axis* indicates the percentage of a specific category of genes in the main category.

All-Unigene sequences of *S. europaea* were aligned on the basis of similarities to the NCBI non-redundant protein databases nr, Swiss-Prot, KEGG and COG (e-value <0.00001). These alignments retrieved proteins with the highest sequence similarity to the *S. europaea* unigenes along with their functional annotations. Among the 109,712 high-quality All-Unigene sequences from *S. europaea*, 50,767 (46.27%) had significant BLAST matches in the NCBI non-redundant protein database and 36,156 (32.96%) aligned to Swiss-prot protein sequence database. Of the group, 20,473 (18.66%) aligned with the KEGG database and were assigned to 120 KEGG pathways (Additional File S4). Most of these unigenes were sorted to metabolic pathways (6,303 unigenes), biosynthesis pathways for secondary metabolites (3,507 unigenes) and pathways involved in plant-pathogen interactions (1,665 unigenes) (Additional File S5). Of All-Unigenes, 33,636 (16.2%) unigenes aligned to the COG database and were classified into 25 functional categories (Figure 2). After Blast2GO analysis, 35,268 (32.15%), 43,713 (39.84%) and 21,334 (19.45%) unigenes were classified into 44 terms from three ontologies involved in biological processes, cellular components and molecular function, respectively (Figure 3).

### Differential genes expression of*S. europaea* between salt-free and salt-treated shoots

**Figure 4:**
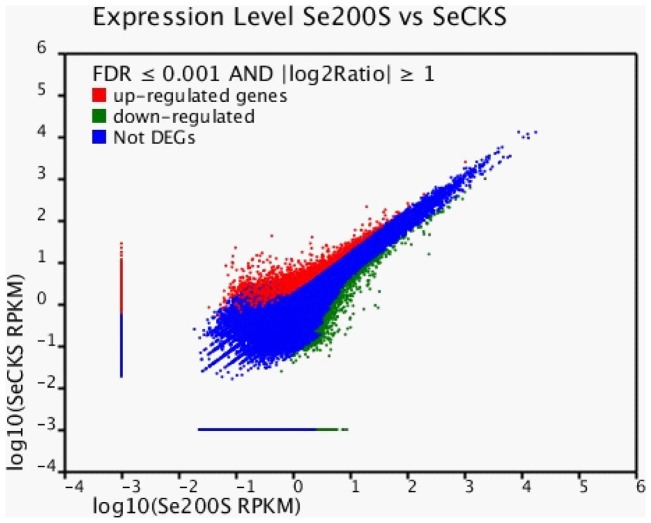
Se200S-vs-SeCKS differentially expressed genes. DEGs were filtered using FDR ≤0.001 and Log2Ratio ≤1 as a threshold. Red spots represent upregulated genes, and green spots indicate downregulated genes. Blue represents those genes that did not show obvious changes between the Se200S and SeCKS samples.

To identify genes with differential expression under different salt conditions, we compared the transcriptome profiles of SeCKS and Se200S. The transcriptome profile of SeCKS was defined as control to get the up- or down-regulated unigenes in Se200S. The results showed that 3,979 out of 109,712 total unigenes were differentially expressed in shoots of *S. europaea* under salt-free and salt-treated conditions, including 2,275 unigenes up-regulated genes (FDR<0.001) and 1,704 down-regulated genes (FDR<0.001). In addition, there were 348 of 2,275 unigenes detected only in Se200S, while 460 of 1,704 unigenes were only expressed in SeCKS (Additional File S6, Figure 4).

Go annotation and statistical analyses demonstrated that differentially expressed genes were classified into three GO ontologies and 35 terms. Of these, 1,469 were in the category of biological processes, 1,972 were in the category of cellular components and 880 were in the category of molecular function (Additional File S7). In the molecular function category, 880 DEGs mapped to seven terms including antioxidant activity, binding, catalytic activity, enzyme regulator activity, molecular transducer activity, protein binding transcription factor activity and transporter activity. For further clarification of gene function, 797 DEGs were enriched in 199 terms, with a *p*-value as good as or better than 1 (Additional File S8). The overwhelming majority of the genes with differential expression between salt-free and salt-treated conditions were related to transmembrane transporter activity (49 genes, or 6.1%), iron ion binding (36 genes, or 4.5%), transition metal ion binding (106 genes, or 13.3%), ion binding (173, or 21.7%), cation binding (166 genes, or 20.8%), metal ion transmembrane transporter activity (9 genes, or 1.1%) and active transmembrane transporter activity (36 genes, or 4.5%). The differentially expressed transporter and ion binding genes are presumed to be important for maintaining and re-establishing the ion homeostasis of the cytoplasm. ATPase-related genes were also differentially expressed with a high frequency and were included in five terms as follows: ATPase activity (36 genes, or 4.5%), ATPase activity, coupled (33 genes, or 4.1%), ATPase activity, coupled to transmembrane movement of substances (17 genes, or 2.1%), ATPase activity, coupled to movement of substances (17 genes, or 2.1%), and ATPase activity, coupled to transmembrane movement of ions and phosphorylative mechanisms (3 genes, or 0.4%). One unigene (Unigene30025_All) classified into the symporter activity term was upregulated dramatically in Se200S and annotated as a Na^+^ symporter family member protein from *Populus trichocarpa*. Three differentially expressed unigenes (Unigene21877_All, Unigene22273_All and Unigene39816_All) were categorised in the antiporter activity term and were identified as a Na^+^/H^+^ antiporter, TT12-2 MATE transporter and a Na^+^/H^+^ exchanger 4, respectively. The discovery of these differentially expressed genes at the whole transcriptome level is expected to clarify the molecular mechanisms of ion absorption and transmembrane transport by *S. europaea*.

Living organisms are complex systems that are flexible and adaptive to their surroundings. At the molecular level, different genes generally cooperate with each other to exercise their biological functions in intricate networks of molecular reactions. In our study, KEGG pathway analysis helped us to understand the biological function of these (DEGs) under salt-free and salt-treated conditions. In this analysis, 1,094 DEGs mapped to 101 pathways (FDR ≤0.05) with most mapping to metabolic pathways (322 DEGs), biosynthesis pathways for secondary metabolites (186 DEGs), pathways involved in plant-pathogen interactions (59 DEGs), protein processing pathways in the endoplasmic reticulum (40 DEGs), starch and sucrose metabolism pathways (30 DEGs) and ABC transporter pathways (6 DEGs) (Additional File S9).

### Quantitative real-time PCR validation

The salt tolerance mechanisms of *S. europaea* are not yet clear. However, it is possible that the survival of this plant depends on the accumulation of high levels of ions in the tissue for the maintenance of osmotic balance, with the accumulation of Na^+^ being higher than other ions under salt treatment. K^+^ and Ca^2+^ uptakes were also promoted by moderate concentrations of NaCl but were reduced at higher salt concentrations [52]. In this research, we isolated many transporter and ion binding genes that were presumed to be effector molecules for coping with salt stress.

**Figure 5:**
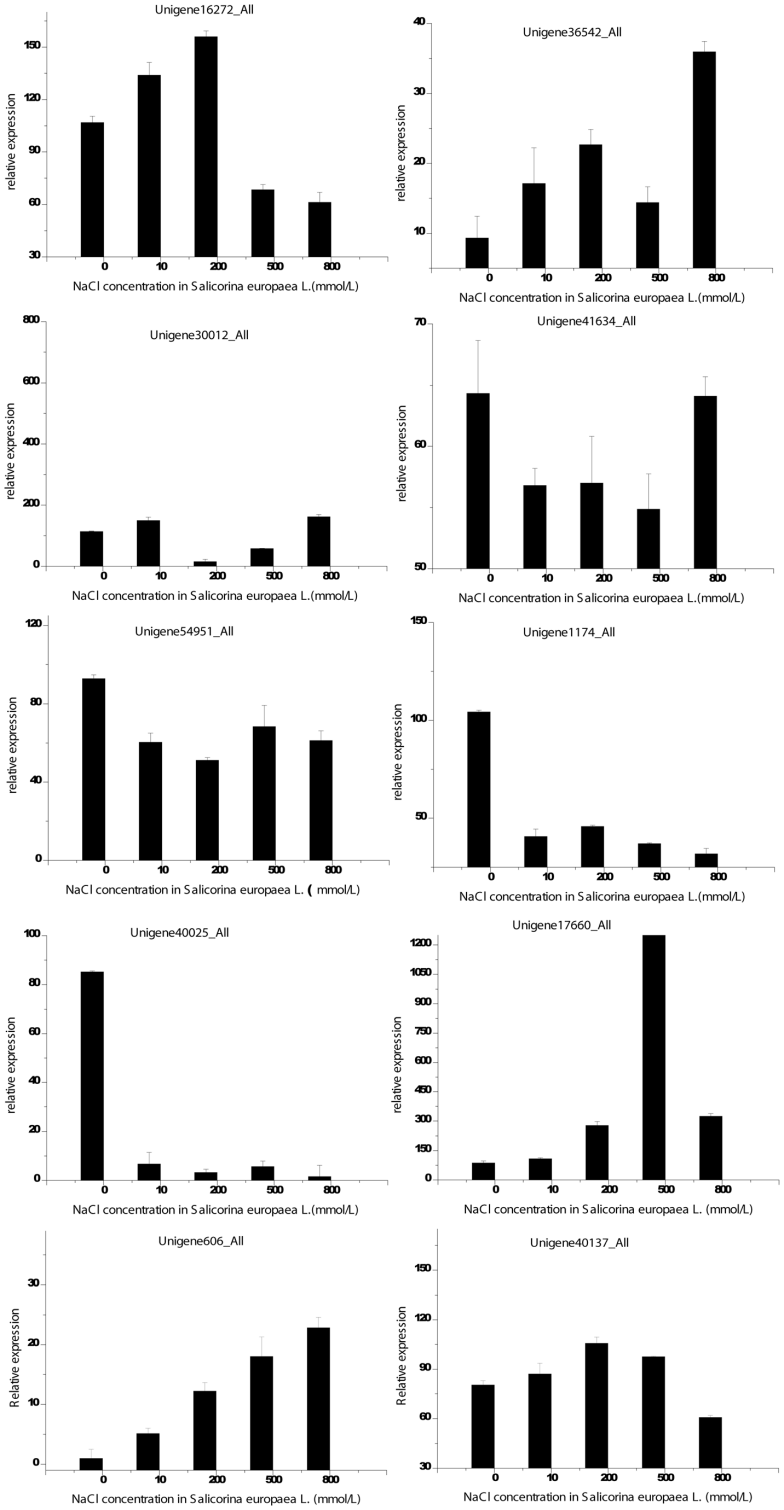
Validation of Transcriptome Profiling expression with QRT-PCR. The transcript abundance from Transcriptome Profiling data is shown above each gene. Relative transcript levels are calculated by real-time PCR using alpha-tubulin as the standard. Three biological replicates were performed, and the data shown are typical results.

Five cDNAs were homologous to HKT genes from *Populus trichocarpa* and *Suaeda salsa*, as determined by protein database searches. HKT genes are reported to be involved in Na^+^ homeostasis under salt stress in several plant species [53]. The sequence analysis of five cDNAs showed reduced similarity to each other at the nucleotide level, and we have speculated that these HKT genes are new members of the HKT family and perform distinct functions for salt adaptation in *S. europaea*. Unigene16272_All is an HKT gene homologous to the hkt1-like gene of *Populus trichocarpa*, a sodium transporter. qPCR results revealed that the expression of this gene was upregulated with increasing salt concentration, whereas it decreased beyond 200 mM NaCl (Figure 5). Different members of the HKT family exhibit different ion selectivity and transport mechanisms [54], [55]. The role of the HKT gene in *S. europaea* is unclear, and further study is being conducted in our laboratory to clarify the function of the different HKT members of *S. europaea* in a saline habitat.

Vacuolar compartmentation of Na^+^ is an important mechanism for salt adaptation in plants. This process is undertaken by vacuolar membrane Na^+^/H^+^ exchangers (NHX) and is driven by the intracellular electrochemical gradient of protons [56], [57]. At the cellular level, Na^+^ accumulation in vacuoles reduces the toxicity of Na^+^ ions in the cytoplasm and in sensitive organelles and also lowers the osmotic potential of the vacuoles to preserve cellular turgor pressure and cell expansion under saline stress. Therefore, the translocation and storage of Na^+^ inside vacuoles of the shoot are thought to be important mechanisms for sustained growth under saline conditions by halophytes such as *S. europaea* [58]. Here, we identified four NHX genes that are homologous to genes in *Arabidopsis thaliana, Mesembryanthemum crystallinum, Chrysanthemum morifolium* and *Oryza sativa*. As determined by qRT-PCR analysis, Unigene36542_All (Na^+^/H^+^ exchanger 4) was upregulated gradually with increasing NaCl concentration, with the exception of 500 mM NaCl (Figure 5). In Arabidopsis, AtNHX4 localises to vacuoles, transports Na^+^ out of the vacuolar lumen and into the cytosol and functions in Na^+^ homeostasis in plant cells [59], [60]. The functions of NHX4 in euhalophytes are still unknown, however, and merit further exploration. We have suggested that Na^+^/H^+^ exchanger 4 plays a crucial role in the maintenance of the normal growth of *S. europaea* under saline conditions by sequestering Na^+^ into vacuoles.

Plants can cope with osmotic stress by synthesising low molecular weight hydrophilic proteins [58] such as osmotin. In *S. europaea*, Unigene30012_All is homologous to an osmotin protein belonging to a member of the PR5 group, and it was differentially expressed in salt-treated and salt-free shoots (Figure 5). Previous studies determined that osmotin up-regulation and overexpression may enhance plant tolerance to NaCl stress [61], [62]. In contrast to glycophytes, the osmotin gene in *S. europaea* was expressed at its lowest level at the highest Na^+^ concentration (200 mM) and, remarkably, increased in expression in response to both low and high Na^+^ treatments below 200 mM (Figure 5). This expression helped to increase cellular turgor pressure at lower Na^+^ levels and improved osmoregulation of the cytoplasm at higher Na^+^ levels. Proline and other amino acids are ubiquitous osmoregulation compounds in plants. The synthesis and transport of these amino acids promote salt tolerance in most plants [63]–[65]. The genes proline transporter (ProT, Unigene41634_All) and amino acid permease (AAP, Unigene54951_All) were identified as homologous to genes in *Populus trichocarpa*. Similar to osmotin, the ProT gene was upregulated in the absence of salt and at the highest salt concentration but remained low in 10–500 mM NaCl. The AAP gene was highly transcribed in salt-free conditions (Figure 5). Previous studies have reported that *ProT* accumulation is increased under salt stress in most plants [64], [66], [67]. This finding suggests that a salt-free environment is deleterious for halophytes and that these plants will synthesise and transport various osmoprotectants to maintain cell turgor pressure under osmotic stress [68], [69]. In addition, the genes vinorine synthase (Unigene1174_All) and salt-induced protein (Unigene40025_All) were dramatically upregulated under salt-free conditions (Figure 5). These results suggest that *S. europaea* is likely to synthesise, transport and accumulate low-molecular weight organic compounds such as osmotin, proline, other amino acids, simple sugars, disaccharides, alkaloid and stress protein in the cytosol and the organelles to improve cellular turgor pressure and cell expansion in the shoots under salt-free conditions.

On the other hand, previous studies showed that NaCl treatments suitable for growth, such as 200 mM NaCl, caused a significant increase in plant biomass in some halophytes, while salt-free and high-salt conditions could decrease plant height and biomass [40], [74]. Plant growth and development are regulated by Gibberellin (GAs), which is modulated by the various genes involved in GAs biosynthesis and deactivation. In particular, GAs biosynthesis is tightly controlled by two gene families that catalyse bioactive GAs formation, the GA 20-oxidases (GA20ox) and the GA 3-oxidases (GA3ox). The GA 2-oxidases (GA2ox), on the other hand, catalyse the deactivation of bioactive GAs. In Arabidopsis, the cellular concentration of bioactive GAs was reduced via an increase in GA2ox7 transcript levels [72], resulting in an accumulation of DELLA, an inhibitor of plant growth [72], [73]. Based on transcriptome sequence analysis of *S. europaea*, we found that several GAs genes were regulated in various ways by 200 mM NaCl. Unigene21299_All and Unigene5033_All are homologues of gibberellin 3-oxidase and gibberellin 20-oxidase from *Populus trichocarpa*, and GA20ox and GA3ox were detected at a higher level than GA2ox (Unigene19515_All) in plants treated with 200 mM salt. In addition, there are two DELLA domain GRAS family transcription factors and DELLA protein GAI that were down-regulated in plants treated with 200 mM salt, albeit at a higher level than in salt-free plants. We propose a model whereby GAs are upregulated in halophytes under appropriate salt concentrations (200 mM) for normal growth of *S. europaea*, accompanied by negative regulation of the growth-repressing DELLA proteins.

Plants protect cells and subcellular systems from salt damage caused by ROS using antioxidant enzymes such as superoxide dismutase (SOD), catalase (CAT), peroxidase (POX) and glutathione reductase [70]. In *S. europaea*, Unigene17660_All is a homologue of a peroxidase from *Spinacia oleracea* that was gradually upregulated after NaCl treatment but decreased in concentration with 800 mM NaCl treatment (Figure 5). According to the results of a previous study, peroxidase activity increases to protect plants against stress [71]. In this study, we identified many differentially expressed genes. However, many have no homology to database sequences and no known function. These proteins may play a critical role in *S. europaea* adaption to saline conditions. To examine the validity of these genes, two genes (Unigene606_All and Unigene40137_All) were selected and qRT-PCR was performed. The analysis showed that the expression of each gene was influenced significantly by different NaCl solutions (Figure 5).

## Conclusions

Totals of 41 million and 39 million clean reads from the tissues Se200S and SeCKS from *S. europaea* shoots were obtained using the Illumina HiSeq™ 2000. The assembled sequences represent a substantial part of the transcriptome of this plant. Transcriptome comparisons identified the DEGs that play significant roles in response to salt-free and salt-treated conditions. These results will facilitate the discovery of specific stress-resistant genes in *S. europaea*, aid the analysis of expression profiles of salt-tolerance-related genes, prompt studies on the molecular mechanisms of salt resistance, and lead to the development of novel plant cultivars through genetic engineering. More importantly, this study creates a new method for large-scale identification of resistance genes from native wild plants and for the conservation of germplasm resources from rare and endangered plants.

## Supporting Information

Additional File S1 **Primers used for experimental validation and gene expression profile analysis.**
(DOCX)

Additional File S2 **Contains:** Additional File S2.z01– **All-Unigene sequences of** ***S. europaea*** **shoots.** Sequences with no gap and with a length longer than 200 bp were selected from the assembly results. Additional File S2.z02**– All-Unigene sequences of** ***S. europaea*** **shoots.** Sequences with no gap and with a length longer than 200 bp were selected from the assembly results. Additional File S2**– All-Unigene sequences of** ***S. europaea*** **shoots.** Sequences with no gap and with a length longer than 200 bp were selected from the assembly results.
(ZIP)

Additional File S3 **Primers and amplification results of 18 unigenes used to confirm the sequencing quality.** The table shows sequences and annealing temperatures (Tm) and production of primers.
(XLS)

Additional File S4 **Functional annotation of All-Unigenes, including GO, COG, and KEGG analysis.** All-Unigene sequences were searched against protein databases (Nr, SwissProt, KEGG, COG, and GO) using BLASTX (E-value ≤10–5).
(ZIP)

Additional File S5 **Summary of All-Unigenes enriched in KEGG pathways.** Pathway ID and gene number are given in the table. The q-value for all of these pathways was less than or equal to 0.05.
(XLSX)

Additional File S6 **Summary and functional annotation of identified DEGs.** Unigenes with an absolute value of |log^2^Ratio| ≥1 and FDR ≤0.001 were identified as DEGs. GO and KEGG analyses of DEGs were based on a cutoff E-value of less than or equal to 10–5.
(XLS)

Additional File S7 **GO categories of DEGs between Salt-Free and Salt-Treated Shoots of** ***S. europaea.*** DEGs were divided into three major categories: molecular function, cellular component and biological process. The gene numbers and gene ID are listed in this file.
(XLS)

Additional File S8 **Summary of DEGs GO Molecular Function.** GO functional classification annotation provides a gene list and gene number for every specific GO term. **Additional File S9 Summary of DEGs enriched in KEGG pathways.** Pathways and backbone gene numbers are given in the table. The q-values for all pathways are less than or equal to 0.05.
(XLSX)

Additional File S9 **Summary of DEGs enriched in KEGG pathways.** Pathways and backbone gene numbers are given in the table. The q-values for all pathways are less than or equal to 0.05.
(XLS)
