# Supplementary material for: Global Transcriptome Profiling of Salicornia europaea L. Shoots under NaCl Treatment
Source: PLoS One. 2013 Jun 25;8(6):e65877. doi: 10.1371/journal.pone.0065877 (PMC3692491; doi:10.1371/journal.pone.0065877)
Supplement: Additional File S1 — Primers used for experimental validation and gene expression profile analysis. (DOCX) [file pone.0065877.s001.docx]

| Unigene ID | Sequence(5’to3’) | Gene discription |
| --- | --- | --- |
| Unigene16272_All | Forward: ATTGGGTTGGTTGTGGTT | Sodium transporter hkt1-like protein [Populus trichocarpa] |
|  | Reverse: CTCTTTTGCCTTTTGCTT |  |
| Unigene36542_All | Forward: TTTCGTGTTTCCTCTGTC | NHX Na+/H+ exchanger 4 [Arabidopsis thaliana] |
|  | Reverse: TCTTGGTTTGGAGTTTGT |  |
| Unigene30012_All | Forward: CCCCCAAAAGTGGAAGAC | osmotin-like protein [Atriplex nummularia] |
|  | Reverse: CCAGGAGGGCAAGAGAAG |  |
| Unigene41634_All | Forward: GATGTCTTAGGGTATGGGTGGA | proline transporter [Populus trichocarpa] |
|  | Reverse: TCTGAGAGAGTGGAAGGATGGA |  |
| Unigene54951_All | Forward: TCACTCAAACCCACACCAAT | amino acid permease [Populus trichocarpa] |
|  | Reverse: GACAGCACTCCCGACCCTAT |  |
| Unigene1174_All | Forward: TGGTTCGTAGAAGAGAAGTAGGG | Vinorine synthase |
|  | Reverse: TAGAAGGGATAAAAGTGGAGGTG |  |
| Unigene40025_All | Forward: GCAGGGAAATGAAGTCGTC | salt-induced protein [Atriplex nummularia] |
|  | Reverse: AAACCTATCAGCGGTGGGC |  |
| Unigene17660_All | Forward: AAGGCTTGAGTGCTGATGA | peroxidase [Spinacia oleracea] |
|  | Reverse: TAAATGCTACGACGGTTGC |  |
| Unigene606_All | Forward: TATTCCTGCTTGCCGTTT | unknow |
|  | Reverse: TGAGCCTTTCACCATCGT |  |
| Unigene40137_All | Forward: CCGAAATCAAAACACGAG | unknow |
|  | Reverse: GGAAAAGAGTAACCGAAG |  |
